# Prevalence of respiratory disease in Irish preweaned dairy calves using hierarchical Bayesian latent class analysis

**DOI:** 10.3389/fvets.2023.1149929

**Published:** 2023-04-13

**Authors:** John D. Donlon, John F. Mee, Conor G. McAloon

**Affiliations:** ^1^School of Veterinary Medicine, University College Dublin, Dublin, Ireland; ^2^Animal and Bioscience Research Department, Teagasc, Animal and Grassland Research Centre, Grange, Dunsany, Meath, Ireland; ^3^Animal and Bioscience Research Department, Teagasc, Moorepark Research Centre, Fermoy, Co. Cork, Ireland

**Keywords:** BRD, pneumonia, Bayesian, calf, thoracic ultrasound, clinical scoring system

## Abstract

**Introduction:**

Bovine respiratory disease (BRD) has a significant impact on the health and welfare of dairy calves. It can result in increased antimicrobial usage, decreased growth rate and reduced future productivity. There is no gold standard antemortem diagnostic test for BRD in calves and no estimates of the prevalence of respiratory disease in seasonal calving dairy herds.

**Methods:**

To estimate BRD prevalence in seasonal calving dairy herds in Ireland, 40 dairy farms were recruited and each farm was visited once during one of two calving seasons (spring 2020 & spring 2021). At that visit the prevalence of BRD in 20 calves between 4 and 6 weeks of age was determined using thoracic ultrasound score (≥3) and the Wisconsin respiratory scoring system (≥5). Hierarchical Bayesian latent class analysis was used to estimate the calf-level true prevalence of BRD, and the within-herd prevalence distribution, accounting for the imperfect nature of both diagnostic tests.

**Results:**

In total, 787 calves were examined, of which 58 (7.4%) had BRD as defined by a Wisconsin respiratory score ≥5 only, 37 (4.7%) had BRD as defined by a thoracic ultrasound score of ≥3 only and 14 (1.8%) calves had BRD based on both thoracic ultrasound and clinical scoring. The primary model assumed both tests were independent and used informed priors for test characteristics. Using this model the true prevalence of BRD was estimated as 4%, 95% Bayesian credible interval (BCI) (1%, 8%). This prevalence estimate is lower or similar to those found in other dairy production systems. Median within herd prevalence varied from 0 to 22%. The prevalence estimate was not sensitive to whether the model was constructed with the tests considered conditionally dependent or independent. When the case definition for thoracic ultrasound was changed to a score ≥2, the prevalence estimate increased to 15% (95% BCI: 6%, 27%).

**Discussion:**

The prevalence of calf respiratory disease, however defined, was low, but highly variable, in these seasonal calving dairy herds.

## 1. Introduction

Bovine respiratory disease (BRD) is one of the major challenges associated with rearing dairy calves internationally (1–3). BRD can be caused by a wide range of both bacterial and viral pathogens (4) and can occur in both clinical and subclinical forms, both the upper and lower respiratory tracts can be affected (5). BRD can reduce calf welfare (6), growth rates, longevity, milk production and reproductive performance (7) and increase antimicrobial usage (8). BRD was found to be the most common (33.4%) cause of mortality in calves (both dairy and beef) aged between 1 and 5 months submitted to regional veterinary laboratories in Ireland (1). However, inherent submission biases in this material means it may not truly reflect on-farm BRD prevalence (9). Understanding the prevalence of BRD would allow for a better estimation of its impact on animal welfare, antimicrobial usage and farm profitability.

Establishing the prevalence of BRD is challenging as currently there is no gold standard ante mortem test for diagnosis of BRD (5). Bayesian Latent Class Analysis (BLCA) can be used to estimate true prevalence on the basis of results from multiple imperfect diagnostic tests (10). In the case of BRD, Bayesian techniques provide the opportunity to integrate our prior knowledge from BRD post-mortem data and other cross sectional studies with data collected using multiple diagnostic modalities in live calves to give an accurate estimate of the true prevalence of BRD. In this work a hierarchical Bayesian model was used, this type of model allows utilization of information about BRD prevalence from the different levels of observation unit i.e., animal, herd, national (11). This allows us to create distributions for both herd level prevalence and within herd prevalence.

Traditionally BRD was diagnosed by veterinarians using thoracic auscultation and clinical examination. It has been shown auscultation has moderate sensitivity (Se) (proportion of calves with BRD that test positive) (Se; 0.63–0.72) (12, 13) and moderate to low specificity (Sp) (proportion of calves without BRD that test negative) (Sp; 0.46-0.53) (12, 13). Currently two BRD diagnostic techniques are commonly used in research studies; thoracic ultrasonography (TUS) (14–20) and clinical respiratory scoring (CRS) (3, 15, 16, 20–22). Given the limitations of each modality, some authors opt to use both TUS and CRS when diagnosing or defining BRD (15), some authors may choose to use the tests in parallel to increase Se while others may choose to use the tests in series to increase Sp.

Thoracic ultrasound (TUS) is a technique that has grown in popularity in recent years in research settings (15–20). It has similar Se (0.60–0.90) (13, 23–25) but superior Sp (0.77–0.95) (13, 23–25) to auscultation (Sp; 0.46–0.53) (12, 13). TUS is not a perfect diagnostic tool. False negative diagnosis may occur if a calf is screened early in the BRD process, if BRD is present without pulmonary consolidation or consolidation which is not observed during screening because it is surrounded by aerated lung (25). False positives can occur if non-BRD lesions such as atelectasis or neoplasm are misidentified (25). BRD is not confined solely to the lung parenchyma it can also include the upper respiratory tract which is not imaged by thoracic ultrasound (14).

A commonly used clinical scoring system is the Wisconsin respiratory scoring system (3, 15–17, 20, 21, 25–29). It assigns certain clinical signs (ocular discharge, nasal discharge, ear position, response to tracheal pinch/presence of cough, rectal temperature) a score from 0 to 3 depending on how significantly they deviate from normal. These scores are then added together and if the animal-level score surpasses a certain threshold a case of BRD is diagnosed. There are numerous published versions and interpretations of the Wisconsin respiratory scoring system (20, 21, 26–29), other scoring systems such as the California score also exist (22). The Se and Sp of clinical scoring varies widely in the literature (Se; 0.48–0.78, Sp; 0.74–0.99) (23–25). Clinical scoring systems can have poor inter-rater agreement as shown by Buckzinski et al. (30). Berman et al. (31) showed there is poor inter-rater agreement in individual clinical signs that make up a clinical scoring system. Because of this, relying solely on clinical scoring systems to estimate prevalence may give an unreliable result.

Accepting these diagnostic limitations, numerous studies internationally have reported the calf-level apparent prevalence of BRD in dairy calves. Dubrovsky et al. (32) estimated a calf-level apparent prevalence of 8.2% on dairy farms in California using the California BRD scoring system. Lago et al. (29) found a BRD apparent calf level prevalence of 14% in Wisconsin during winter using the Wisconsin respiratory scoring system. Buczinski et al. (18) found a herd-level prevalence of lung consolidation on TUS ≥3 cm of 8% in the summer and 15% in the winter in dairy farms in Quebec. Van Leenen et al. (15) found a prevalence of 20.2% of calves with a CRS ≥5 and 16.1% of calves with TUS ≥6 cm. None of the sighted studies used a gold standard test or used any statistical method to improve the accuracy of the prevalence estimation. In most cases these calves were managed in all-year-round calving, confinement systems or veal units which have contrasting management systems to seasonal calving dairy herds.

Spring calving dairy herds are managed to synchronize the highest demand for feed (peak lactation) with the highest availability of grazed grass (33). This production system allows for farms to maintain higher levels of profitability due to the lower production costs of grass (34). It is recommended that 90% of the milking herd be calved within the first 6 weeks of the calving season (35). The compact nature of the calving season places increased strain on labor and infrastructure during the calf rearing period. Calf care has been estimated to only make up 14% of a farmers time budget during spring (34), meaning reduced time for disease monitoring and important management practices (cleaning, bedding, feeding). Group housing of calves is a common practice in Ireland as it is regarded as being more labor efficient (34), an average group size of 11 calves was found in work by Barry et al. (36). Ollivett (37) recently reviewed group housing and BRD and showed some evidence, although not conclusive, that grouping in particular larger groups of calves had a higher risk of BRD. The lack of time that can be allocated to calf management and the common use of large group housing are factors that the authors hypothesis might lead to an increased prevalence of BRD in Irish preweaning calves.

There are no studies that estimate the prevalence of BRD in seasonal systems and there are no studies that estimate the true prevalence of BRD when taking into account the lack of a gold standard test. Hence the aim of this study was to estimate the calf- and farm- level true prevalence of BRD using two imperfect diagnostic tests and a hierarchical Bayesian latent class model.

## 2. Materials and methods

### 2.1. Ethics

This study was approved by University College Dublin, Animal Research Ethics Committee and the Health Products Regulatory Authority (V016/2020Q1).

### 2.2. Herd selection

Herds were recruited to this study using a database previously used in a national study of contract-reared vs. home-reared heifers (38). The control farms in that study, i.e., dairy farmers who home-reared their heifers, were recruited *via* a letter that had been sent to randomly selected spring calving dairy farmers in the Republic of Ireland that were in the HerdPlus database of the Irish Cattle Breeding Federation (ICBF). The dairy farmers who home-reared their heifers and enrolled in that study (*n* = 56) were sent a letter describing the current research project and asked if they were interested to contact a member of the research team. No geographical limit was put on recruitment, In total, 40 farms were recruited.

### 2.3. Calf enrolment

Each farm was visited once during either spring 2020 or spring 2021; the sampling period had to be split over 2 years due to the COVID 19 pandemic. In 2020 (February and March), 28 farms were visited and 547 calves were examined. In 2021 (February and March), 12 farms were visited and 240 calves were examined. In Ireland spring is considered to start in March (39)^1^; the majority of farm visits were conducted in March. At each visit, 20 dairy calves, of either sex, were recruited for examination. A separate aim of this work was to investigate the interaction between housing environment and BRD prevalence, a power calculation carried out determined that 20 calves were required on each farm for this work. Calves were selected by age. Calves between 4 and 6 weeks of age were randomly selected but if a farm did not have 20 calves between 4 and 6 weeks of age then calves older than 6 weeks but not weaned were chosen preferentially. If none of the latter were available, the calves closest to 4 weeks were chosen instead. Four to six weeks has previously been identified as the age at which the peak prevalence of BRD is observed (17, 29). A preference was made to sample all calves in the same pen as opposed to a random selection from multiple pens. All calves that were enrolled had their estimated weight recorded using a weigh band (Volac International Limited, Hertfordshire, UK).

### 2.4. Clinical respiratory scoring

The Wisconsin respiratory scoring system was used as described by McGuirk and Peek (21). The CRS was carried out before the TUS was performed. The CRS was performed by 10 operators throughout the study with each operator directly supervised by the first author and each had access to a reference scoring chart; logistically it was not possible to have a single operator conduct all of the scoring. Blinding of results of the CRS to the first author who carried out the TUS was not possible in this work. Nasal and ocular discharge, ear position, presence of a cough/response to a tracheal pinch and rectal temperature were all scored on a scale from 0 to 3 with 0 being normal and 3 being severely abnormal. Calves were considered to be positive when they had a CRS score ≥5 or if they had two or more of the individual scores that were ≥2.

### 2.5. Thoracic ultrasound scoring

All of the TUS was performed by a single operator (first author) using a portable linear rectal ultrasound scanner set at a depth of 7 cm and frequency of 7 MHz (Easi-Scan Go, IMV Technology Ltd.). A 70% isopropyl alcohol solution was sprayed onto the unclipped hair on both sides of the thorax. The technique described by Olivett and Buczinski (14) was used. In brief the scan was started at the 10^th^ intercostal space on the left-hand side and the probe was moved in a dorso-ventral direction down each intercostal space to the 2^nd^ on the left hand side. On the right hand side a similar technique was used but to allow for imaging of the most cranial portion of the lung lobe, the right fore-limb was drawn cranially by an assistant to allow ease of imaging of the 1^st^ and 2^nd^ intercostal space.

The entire lung field was examined and assigned a score from 0 to 5 as previously described by Ollivett and Buczinski (14): 0 = normal aerated lung with no consolidation and one to few comet tail artifacts; 1 = diffuse small comet tail artifacts without consolidation; 2 = isolated patches of consolidation, 3 = consolidation of a full lung lobe, 4 = consolidation of 2 lung lobes, 5 = consolidation of 3 or more lung lobes. A TUS score ≥3 was considered positive.

### 2.6. Types of BRD

The BRD syndrome was classified into three separate presentations as defined by Ollivett and Buczinski (14) using the CRS and TUS results; (1) Upper respiratory tract disease (URTD): defined by a significant clinical score (≥5) in the absence of significant lesions identified by TUS(< 3), (2) Subclinical pneumonia/lower respiratory tract disease (LRTD): defined by a non-significant clinical score (< 5) with significant lesions identified on TUS (≥3) and (3) Clinical respiratory disease (CRD): defined by both a significant clinical score (≥5) and significant lesions identified on TUS (≥3).

### 2.7. Data management

Prior to the visit animal records were downloaded from the ICBF database and uploaded to a file maker pro application which allowed for identification of calves based on age and subsequent recording of CRS and TUS scores. After the visits the data was exported as a CSV file which was then imported into R version 4.0.0 (https://www.r-project.org/) for further data processing and statistical analysis. All data manipulation, visual presentation and statistical analysis was conducted using “tidyverse” (40) and “rjags” (41) packages. Calves with missing CRS or TUS data were removed from the dataset prior to modeling.

### 2.8. Statistical analysis

Prevalence of calf-level BRD was estimated with a hierarchical Bayesian latent class model extended from that proposed by Branscum et al. (42) and Hanson et al. (43). There were three main assumptions in this model: (1) Se and Sp of the tests were constant across all herds, (2) the results of each test were considered conditionally independent of each other, and (3) prevalence of disease varied across herds, a proportion of which were disease free, and within affected herds, the within-herd prevalence of diseased animals followed a logit-normal distribution.

For the *j*-th herd, the *n*_*j*_ observations gave the data vector for the joint test results *P*_11_, *P*_10_, *P*_01_, *P*_00_. Where the *P*_11_ is the number of animals from the *j*th herd that test positive on TUS and CRS and *P*_10_ is the number that test positive to TUS and negative to CRS and so on. *P*_11_ to *P*_*i*_ are assumed to have multinomial sampling distributions. We assume that calves examined are an unbiased sample of the calves in the herd.


$$\begin{array}{l} {\text{y}}_{j}\sim \text{multinomial}\left(n_{\text{j}},\left(p_{11j},p_{10j},p_{01\text{j}},p_{00j}\right)\right) \end{array}$$

The multinomial cell probabilities are given by:


$$\begin{array}{c} {\text{P}}_{\text{11j}}=\pi_{\text{j}}\times \text{S}{\text{e}}_{\text{TUS}}\times \text{S}{\text{e}}_{\text{CRS}}+\;\left(\text{1}-\pi_{\text{j}}\right)\;\times \;\left(\text{1}-\text{S}{\text{p}}_{\text{TUS}}\right)\\ \;\times \;\left(\text{1}-\text{S}{\text{p}}_{\text{CRS}}\right) \end{array}$$

$$\begin{array}{c} {\text{P}}_{10\text{j}}=\pi_{\text{j}}\times \text{S}{\text{e}}_{\text{TUS}}\times \;\left(1-\text{S}{\text{e}}_{\text{CRS}}\right)\;+\;\left(1-\;\pi_{\text{j}}\right)\;\times \;\left(\text{1}-\text{S}{\text{p}}_{\text{TUS}}\right)\\ \;\times \text{S}{\text{p}}_{\text{CRS}}\; \end{array}$$

$$\begin{array}{c} {\text{P}}_{0\text{1j}}=\pi_{\text{j}}\times \;\left(\text{1}-\text{S}{\text{e}}_{\text{TUS}}\right)\;\times \;\text{S}{\text{e}}_{\text{CRS}}\;+\;\left(\text{1}-\pi_{\text{j}}\right)\;\times \;\text{S}{\text{p}}_{\text{TUS}}\\ \times \;\left(\text{1}-\text{S}{\text{p}}_{\text{CRS}}\right)\; \end{array}$$

$$\begin{array}{c} {\text{P}}_{00\text{j}}=\pi_{\text{j}}\times \;\left(\text{1}-\text{S}{\text{e}}_{\text{TUS}}\right)\;\times \;\left(\text{1}-\text{S}{\text{e}}_{\text{CRS}}\right)\;+\;\left(\text{1}-\pi_{\text{j}}\right)\\ \;\times \text{S}{\text{p}}_{\text{TUS}}\times \text{S}{\text{p}}_{\text{CRS}} \end{array}$$

where π_*j*_ is the prevalence in the *j*-th herd and Se_TUS_, Se_CRS_, Sp_TUS_, Sp_CRS_ are the sensitivities and specificities of TUS and CRS, respectively.

We chose to model within herd-prevalence using the logit-normal approach as used by Yang et al. (44). A range of methods have been used to model within herd prevalence in these models. Previous work from our group has shown some advantages to this approach compared with, for example, a beta hyperprior approach (45). These include facilitation of increased variation of within herd prevalence while still retaining a distribution shape that is more reflective of the likely distribution (45).

The true within-herd prevalence in each herd (π_*j*_) was modeled as a product of herd-level prevalence (*h*_*j*_) and the ‘conditional' within-herd prevalence (ψ_*j*_). The herd-level prevalence was modeled using a Bernoulli distribution with mean μ representing the proportion of herds that were disease free. The conditional within-herd prevalence was the within-herd true prevalence in affected herds only and was modeled using an intercept only random effect logistic regression (44) where α was the intercept, and ε_*j*_ was the farm level random effect.


$$\begin{array}{r} \pi_{\text{j}}\;\sim {\text{h}}_{\text{j}}\times \psi_{\text{j}}\\ \text{logit}\left(\psi_{\text{j}}\right)\;<-\alpha +\varepsilon_{\text{j}}\\ {\text{h}}_{\text{j}}\sim \;\text{Bernoulli}\left(\mu \right)\\ \varepsilon_{\text{j}}\sim \;\text{normal}\left(0,1/\tau \right)\\ \tau \sim \;\text{gamma}\left(10,10\right)\\ \mu \;\sim \;\text{beta}\left(1,1\right) \end{array}$$

Se and Sp of each test were modeled using beta distributions:


$$\begin{array}{r} \text{S}{\text{e}}_{\text{CRS}}\sim \;\text{beta}\left(\text{alph}{\text{a}}_{\text{Se CRS},},\text{bet}{\text{a}}_{\text{Se CRS}}\right)\\ \text{S}{\text{e}}_{\text{TUS }}\sim \;\text{beta}\left(\text{alph}{\text{a}}_{\text{Se TUS }},\text{bet}{\text{a}}_{\text{Se TUS},}\right)\\ \text{S}{\text{p}}_{\text{CRS}}\sim \;\text{beta}\left(\text{alph}{\text{a}}_{\text{Sp CRS},},\text{bet}{\text{a}}_{\text{Sp CRS}}\right)\\ \text{S}{\text{p}}_{\text{TUS }}\sim \;\text{beta}\left(\text{alph}{\text{a}}_{\text{Sp TUS}},\text{bet}{\text{a}}_{\text{Sp TUS}}\right) \end{array}$$

These beta distributions were used to specify the priors for the Se and Sp of TUS and CRS respectively.

Posterior inferences for each parameter (Se_CRS_, Se_TUS_, Sp_CRS_, Sp_TUS_, π__*j*__, α, ε_*j*_and *h*_*j*_) were obtained using JAGS called from R statistical software using the “rjags” package (41). Markov chains ran for 15,000 iterations after a burn in period for 5,000 iterations. Convergence of the Markov chains was assessed by visual assessment of Markov chain and autocorrelation plots, and by running multiple (*n* = 2) chains from dispersed starting values (e.g., 0.05 and 0.95 for variables bounded between 0 and 1). Initially autocorrelation was observed for some sample parameters, therefore chains were thinned by 10 for inference.

The calf level prevalence was then calculated using α and *h*_*j*_:


$$\begin{array}{l} h_{j}\times \frac{e^{\alpha }}{\left(1+e^{\alpha }\right)} \end{array}$$

The prevalence of BRD within affected herds was calculated by:


$$\begin{array}{l} \frac{e^{\alpha }}{\left(1+e^{\alpha }\right)} \end{array}$$

#### 2.8.1. Model priors: test characteristics

To estimate the Se and Sp of clinical scoring and thoracic ultrasound, a systematic literature review was conducted for the priors used in this analysis. In total, 4 papers were found that fulfilled the criteria for TUS (13, 23–25) and 3 papers were found that fulfilled the criteria for CRS (23–25) (see Supplemental material for search strings, inclusion criteria and results). A mean Se for TUS was estimated at 0.73 (0.58, 0.86) and a mean Sp of 0.88 (0.83, 0.93) was estimated. A mean Se 0.66 (0.40, 0.87) and Sp 0.86 (0.72, 0.91) was estimated for clinical scoring. This information was used to produce the priors for both TUS and CRS using the betaExpert function in R [package “prevalence” (46)].

#### 2.8.2. Model priors: prevalence

Based on discussions with the co-authors we used a prior for prevalence in affected herds with a mean of 0.1 and were 95% confident that true prevalence was < 0.5. The corresponding alpha distribution was selected as normal (−2.4, 0.3), this was calculated using Excel (Microsoft). A gamma distribution of (10, 10) was used as the prior for tau. The gamma distribution equates to the variance of the logit of normal distribution and considering our priors used, this allowed for the within-herd prevalence to vary from 0 to 100%.

#### 2.8.3. Sensitivity analysis

As part of the sensitivity analysis, several measures were undertaken: the final model was checked by changing the prior information of both TUS and clinical scoring Se and Sp to non-informative beta (1, 1) distributions while retaining prior distributions for alpha, tau and mu. A case definition of TUS ≥3 was used for the primary models while a case definition of TUS ≥2 was used for subsequent models. A gamma distribution of (10, 10) was used in the primary model with it being varied to (1, 1) and (0.1, 0.1). Finally, the analysis was also conducted assuming conditional dependence between detection methods. Assuming tests are conditionally independent implies that the association between TUS and CRS results are accounted for only by the latent class and no other variable. In contrast, assuming test dependency implies that test outcomes are influenced by other latent variables, other than the latent class of concern, that are common to both tests (TUS and CRS). In this case, dependency between diagnostic tests was modeled as described by Dendukuri and Joseph (47):


$$\begin{array}{l} {\text{P}}_{11\text{j}}=\pi_{\text{j}}\times \;\left({\text{Se}}_{\text{TUS}}\times {\text{ Se}}_{\text{CRS}}+{\text{ CovSe}}_{\text{TUSCRS}}\right)\;+\;(1\;-\pi_{\text{j}})\;\\ \;\times \;\left(\left(1\;-{\text{ Sp}}_{\text{TUS}}\right)\;\times \;\left(1-{\text{Sp}}_{\text{CRS}}\right)\;+{\text{ CovSp}}_{\text{TUSCRS}}\right)) \end{array}$$

$$\begin{array}{l} {\text{P}}_{10\text{j}}\\ \;=\pi_{\text{j}}\times \;({\text{Se}}_{\text{TUS}}\times \;(\text{1}-{\text{Se}}_{\text{CRS}})-{\text{CovSe}}_{\text{TUSCRS}})\;+\;(\text{1}-\;\pi_{\text{j}})\\ \;\times \;((\text{1}-{\text{Sp}}_{\text{TUS}})\;\times {\text{Sp}}_{\text{CRS}}-{\text{CovSp}}_{\text{TUSCRS}})\; \end{array}$$

$$\begin{array}{l} {\text{P}}_{01\text{j}}\\ \;=\pi_{\text{j}}\times \;((\text{1}-{\text{Se}}_{\text{TUS}})\;\times \;{\text{Se}}_{\text{CRS}}-{\text{CovSe}}_{\text{TUSCRS}})\;+\;(\text{1}-\pi_{\text{j}})\\ \;\times \;({\text{Sp}}_{\text{TUS}}\times \;(\text{1}-{\text{Sp}}_{\text{CRS}})\;-{\text{CovSp}}_{\text{TUSCRS}})\; \end{array}$$

$$\begin{array}{c} {\text{P}}_{00\text{j}}=\pi_{\text{j}}\times \;\left(\left(1-\text{S}{\text{e}}_{\text{TUS}}\right)\;\times \;\left(1-\text{S}{\text{e}}_{\text{CRS}}\right)\;+\text{CovS}{\text{e}}_{\text{TUSCRS}}\right)\\ \;+\;\left(1-\pi_{\text{j}}\right)\;\times \;\left(\text{S}{\text{p}}_{\text{TUS}}\times \text{S}{\text{p}}_{\text{CRS}}+\text{CovS}{\text{p}}_{\text{TUSCRS}}\right) \end{array}$$

The covariance between the two tests were estimated below:


$$\begin{array}{l} \eta {\text{CovSe}}_{\text{TUSCRS}}\\ \;=\text{ max}\left(-\left(1-{\text{Se}}_{\text{TUS}}\right)*\left(1-{\text{Se}}_{\text{CRS}}\right),\;-{\text{Se}}_{\text{TUS}}*{\text{Se}}_{\text{CRS}}\right)) \end{array}$$

$$\begin{array}{l} \beta \text{CovS}{\text{e}}_{\text{TUSCRS}}=\text{min}\left(\text{S}{\text{e}}_{\text{TUS}}*\left(1-\text{S}{\text{e}}_{\text{CRS}}\right),\text{S}{\text{e}}_{\text{CRS}}*\left(1-\text{S}{\text{e}}_{\text{TUS}}\right)\right) \end{array}$$

$$\begin{array}{l} \gamma \text{Co}{\text{v}}_{\text{SpTUSCRS}}\\ \;=\text{max}\left(-\left(1-\text{S}{\text{p}}_{\text{TUS}}\right)*\left(1-\text{S}{\text{p}}_{\text{CRS}}\right),\;-\text{S}{\text{p}}_{\text{TUS}}*\text{S}{\text{p}}_{\text{CRS}}\right) \end{array}$$

$$\begin{array}{l} \Delta \text{CovS}{\text{p}}_{\text{TUSCRS}}\\ \;=\text{min}\left(\text{S}{\text{p}}_{\text{TUS}}*\left(1-\text{S}{\text{p}}_{\text{CRS}}\right),\text{S}{\text{p}}_{\text{CRS}}*\left(1-\text{S}{\text{p}}_{\text{TUS}}\right)\right) \end{array}$$

$$\begin{array}{l} \text{CovS}{\text{e}}_{\text{TUSCRS}}\sim \;\text{U}\left(\eta \text{CovS}{\text{e}}_{\text{TUSCRS}},\;\beta \text{CovS}{\text{e}}_{\text{TUSCRS}}\right) \end{array}$$

$$\begin{array}{l} \text{CovS}{\text{p}}_{\text{TUSCRS}}\sim \;\text{U}\left(\gamma \text{CovS}{\text{p}}_{\text{TUSCRS}},\Delta \text{CovS}{\text{p}}_{\text{TUSCRS}}\right) \end{array}$$

A uniform distribution was selected between these 2 bounds assuming an equivalent probability throughout this the range of scores.

## 3. Results

### 3.1. Farm descriptions

**Figure 1:**
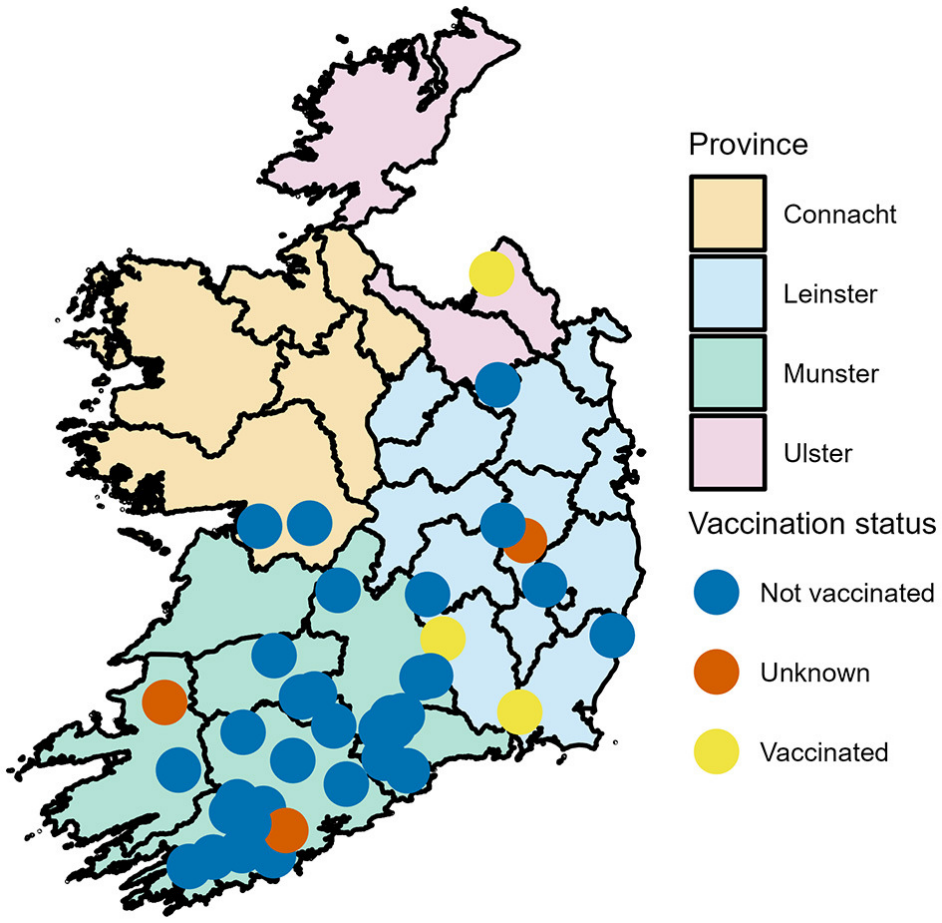
Geographical distribution of dairy farms enrolled in a calf BRD prevalence study across the Republic of Ireland-Locations have been jitter to mask actual location.

The 40 dairy farms recruited all had spring calving herds ranging from 70 to 480 cows, with a median of 145. Figure 1 shows the distribution of recruited farms across Ireland and their vaccination status. The Province of Munster had the majority of farms (*n* = 29, 72.5%) with county Cork having the majority of farms within Munster (*n* = 15).

All farms housed preweaned calves indoors, natural ventilation was utilized in the majority of farms (*n* = 38, 95%) with positive pressure ventilation in use on one farm (2.5%) and negative pressure ventilation in use on one farm (2.5%). Straw bedding was used in the majority of farms (*n* = 38, 95%), one farm used rushes (2.5%), one farm used woodchip (2.5%). All calves were housed in group pens with group sizes varying between 5 and 45 calves. The median pen size in this work was 18 calves. The median number of pens that a calf moves through before weaning was 2 with a range of 1 to 4.

Eighteen farms (45.0%) fed colostrum from only the calves dam, 11 farms (27.5%) fed pooled colostrum without any exclusion criteria, 2 farms (5.0%) used pooled colostrum from selected cows. The remaining farms did not have clear colostrum management policies in place. The median volume of colostrum fed was 3 L. Fifteen farms fed only milk replacer (37.5%), 10 farms fed only whole saleable milk (25.0%), the remaining farms fed some combination of milk replacer and whole saleable milk, of those, 6 (15.0%) indicated that they fed some amount of non-saleable milk. Twenty-eight farms (70.0%) used teat feeders for feeding, 10 (25.0%) of farms used automatic milk feeders and two farms (5.0%) fed calves using buckets.

### 3.2. Calf descriptions

In total, 787 calves were examined. Twenty calves were sampled on each farm with the exception of two farms where 6 and 21 calves were sampled. The median age of the calves was 34 days (range 10–58 days). The majority of calves examined were female (649, 82.5%) and 137 (17.4%) male with one calf's sex unrecorded. Holstein Friesian was the most common calf breed (*n* = 320, 40.7%) followed by Holstein x Friesian x Jersey (*n* = 165, 21.0%), all other dairy breeds made up 26.7% (*n* = 210) and dairy beef crosses accounted for 11.7% (*n* = 92). The median calf body weight was 59 kg (range 31–108 kg). Calves were not vaccinated against BRD on 34 farms, were vaccinated on 3 farms and on 3 farms the calves had unclear vaccination status as can be seen in Figure 1.

### 3.3. Clinical respiratory score

**Figure 2:**
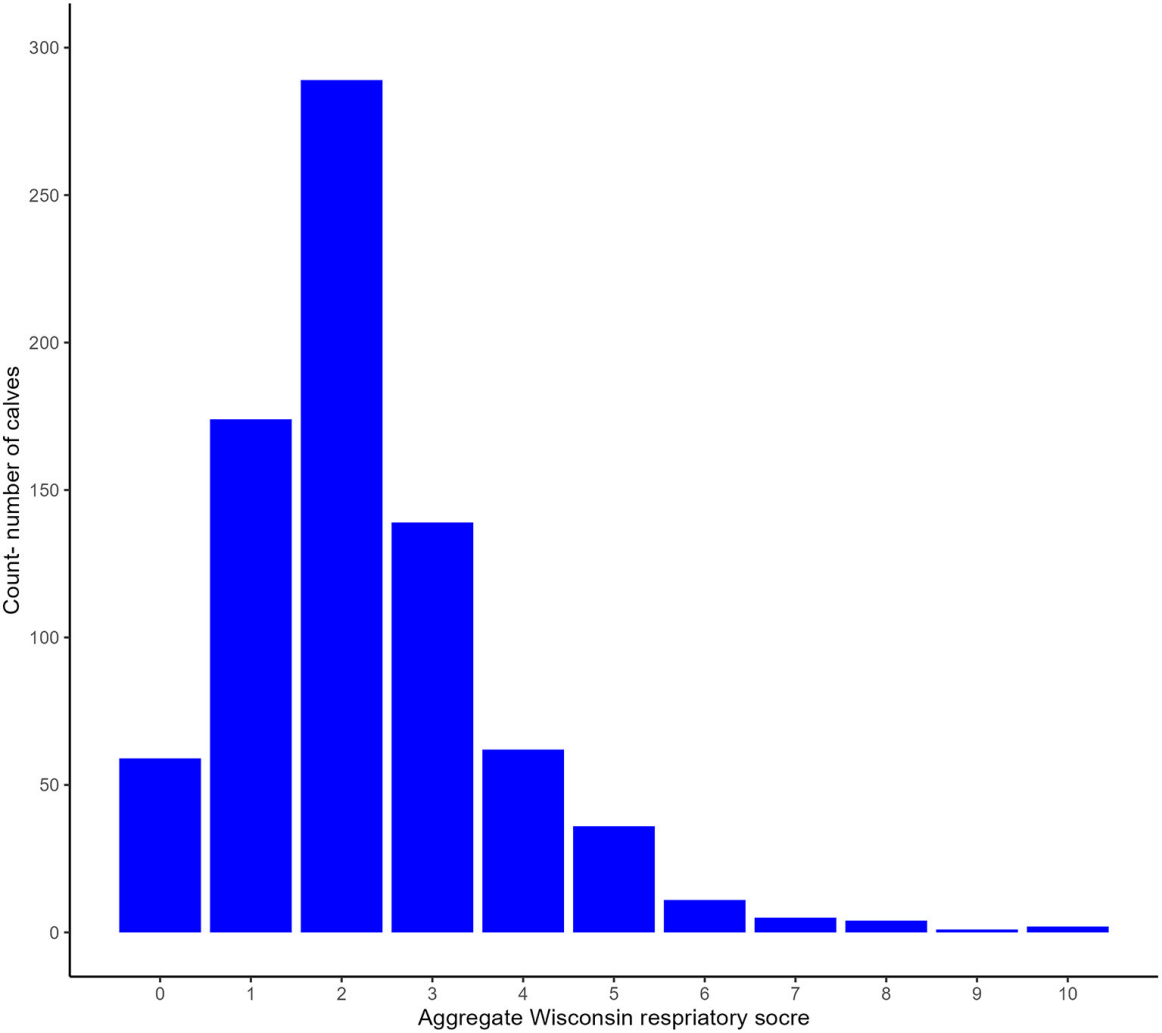
Histogram of the frequency of each aggregate Wisconsin Respiratory score.

The median CRS of all calves was 2 (range 0–10). Table 1 shows the frequency of each of the different clinical scores and Figure 2 is a histogram showing the frequency of each of the aggregate scores. Seventy two calves (9.15%) were positive according to CRS. The median rectal temperature of all calves was 38.9°C (range: 37°C−40.5°C).

**Table 1:** Frequency of occurrence (%, no. calves) of each score for each of the components of the Wisconsin respiratory score.

**Score**	**Cough**	**Nose**	**Eye**	**Ear**	**Temperature score**
0	85.7% (*n* = 675) (no cough)	84.6% (*n* = 666) (normal, serous discharge)	68.1% (*n* = 536) (normal eye)	99.1% (*n* = 780) (normal ear)	13.6% (*n* = 107) (C**°** 37.8–38.2)
1	7% (*n* = 55) (induced single cough)	12.2% (*n* = 96) (small amount of unilateral, cloudy discharge)	30.2% (*n* = 238) (mild ocular discharge)	0.1% (*n* = 1) (ear flicking)	31.4% (*n* = 247) (C**°** 38.3–38.8)
2	5.2% (*n* = 41) (induced repeated coughs or occasional spontaneous)	2.9% (*n* = 23) (bilateral, cloudy, or excessive mucus)	1.4% (*n* = 11) (moderate bilateral ocular discharge)	0.38% (*n* = 3) (slight unilateral ear drop)	48% (*n* = 378) (C**°** 38.9–39.4)
3	1.9% (*n* = 15) (repeated spontaneous coughing)	0.1% (*n* = 1) (copious, bilateral mucopurulent nasal discharge)	0.25% (*n* = 2) (heavy ocular discharge)	0.1% (*n* = 1) (severe head tilt, or bilateral ear droop)	6.9% (*n* = 54) (C**°**> 39.4)
NA	0.1% (*n* = 1)	0.1% (*n* = 1)	0% (*n* = 0)	0.25% (*n* = 2)	0.1% (*n* = 1)

### 3.4. Thoracic ultrasound score

**Figure 3:**
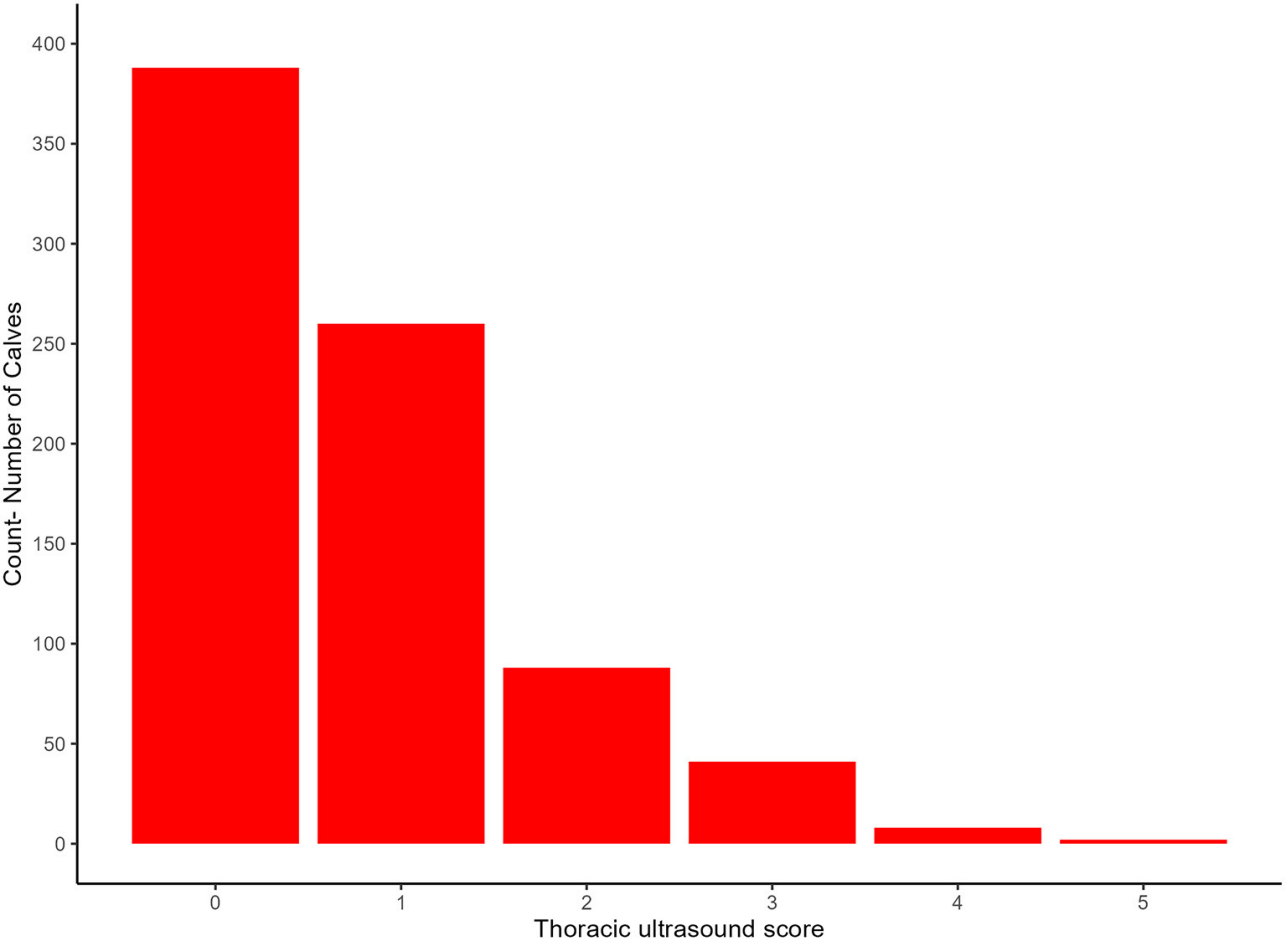
Frequency of each of the thoracic ultrasound scores observed.

The median TUS score of all calves was 1 (range 0–5). Figure 3 shows a summary of the frequency of each of the TUS scores. Fifty one calves (6.48%) had a TUS score ≥3.

### 3.5. Year

In 2020 thirty six of the 547 (6.6 %) calves had a TUS score ≥3 and fifty three of the 547 (9.7%) had CRS ≥ 5. In 2021 fifteen of the 240 (6.3%) calves had a TUS score ≥3 and nineteen of the 240 (7.9%) had a CRS ≥ 5.

### 3.6. BRD subtypes

Upper respiratory tract disease was the most commonly identified type of BRD (CRS +, TUS –) in 58 calves, (53.2% of calves with BRD). Subclinical pneumonia (CR S –, TUS +) was identified in 37 calves (34.0% of BRD cases). Clinical pneumonia (CRS +, TUS +) was identified in 14 calves (12.8% of BRD cases). Table 2 provides a detailed breakdown of the BRD subtypes observed.

**Table 2:** Summary of the BRD subtypes observed.

**BRD subtype**	**TUS** ≥**3**		**TUS** ≥**2**	
	**No. calves**	**%**	**No. calves**	**%**
CRS –, TUS –	678	86.15	603	76.62
CRS +, TUS –	58	7.37	45	5.72
CRS –, TUS +	37	4.70	112	14.23
CRS +, TUS +	14	1.78	27	3.43

A CRS case was considered a score ≥5 or two scores ≥2 while the TUS case definition was either case ≥2 or ≥3. (Healthy: TUS –, CRS –) (Upper respiratory tract disease: TUS –, CRS +), (Lower respiratory tract disease: TUS +, CRS –), (Clinical respiratory disease: TUS +, CRS +).

### 3.7. Bayesian latent class analyses

Convergence was assessed visually using autocorrelation trace plots, initially some auto correlation was detected so the model was thinned by 10. The PSRF values for all monitored variables were < 1.0038 indicating adequate convergence. The effective sample size for each variable ranged from 7,119 to 20,324.

All the results of the final model, as well as the various other models, can be found in Table 3. Median calf-level prevalence of BRD was 4% (95% BCI; 0%, 8%) in the primary model. However median prevalence estimates varied from 4% to 9% in the models using TUS score ≥3 as case definition. In the models where TUS score ≥2 was used as case definition, median prevalence varied between 15% and 25%.

**Table 3:** The median prevalence and Bayesian credible interval (95% BCI) for each of the models undertaken as part of the sensitivity analysis, TUS and CRS and gamma priors, the TUS case definition and model dependency.

**Case definition**	**Gamma prior**	**TUS-prior beta**		**CRS-prior beta**		**Prevalence [median (95% BCI)]**	
		**Se**	**Sp**	**Se**	**Sp**	**Independent models**	**Co-dependent models**
TUS ≥ 2	(10, 10)	(27.6, 10.7)	(89.3, 8.4)	(9.8, 5.6)	(138.8, 18.5)	0.15 (0.06, 0.27)	0.14 (0.07, 0.21)
	(1, 1)	(27.6, 10.7)	(89.3, 8.4)	(9.8, 5.6)	(138.8, 18.5)	0.17 (0.08, 0.27)	0.15 (0.08, 0.22)
	(0.1, 0.1)	(27.6, 10.7)	(89.3, 8.4)	(9.8, 5.6)	(138.8, 18.5)	0.18 (0.09, 0.27)	0.15 (0.09, 0.21)
	(10, 10)	(1, 1)	(1, 1)	(9.8, 5.6)	(138.8, 18.5)	0.20 (0.06, 0.36)	0.13 (0.04, 0.26)
	(10, 10)	(27.6, 10.7)	(89.3, 8.4)	(1, 1)	(1, 1)	0.25 (0.14, 0.38)	0.17 (0.10, 0.26)
TUS ≥ 3	(10, 10)^*^	(27.6, 10.7)^*^	(89.3, 8.4)^*^	(9.8, 5.6)^*^	(138.8, 18.5)^*^	0.04 (0.01, 0.08)^*^	0.05 (0.01, 0.1)
	(1, 1)	(27.6, 10.7)	(89.3, 8.4)	(9.8, 5.6)	(138.8, 18.5)	0.04 (0.01, 0.09)	0.06 (0.01, 0.11)
	(0.1, 0.1)	(27.6, 10.7)	(89.3, 8.4)	(9.8, 5.6)	(138.8, 18.5)	0.04 (0.01, 0.09)	0.07 (0.02, 0.12)
	(10, 10)	(1, 1)	(1, 1)	(9.8, 5.6)	(138.8, 18.5)	0.09 (0.02, 0.19)	0.08 (0.03, 0.16)
	(10, 10)	(27.6, 10.7)	(89.3, 8.4)	(1, 1)	(1, 1)	0.07 (0.01, 0.15)	0.09 (0.04, 0.14)

^*^Denotes the primary model.

In the final model, the median herd prevalence within affected herds was 10% (95% BCI; 1%, 23%), the median prevalence within affected herds varied from 3% to 22% in models using TUS score ≥3 as case definition.

**Figure 4:**
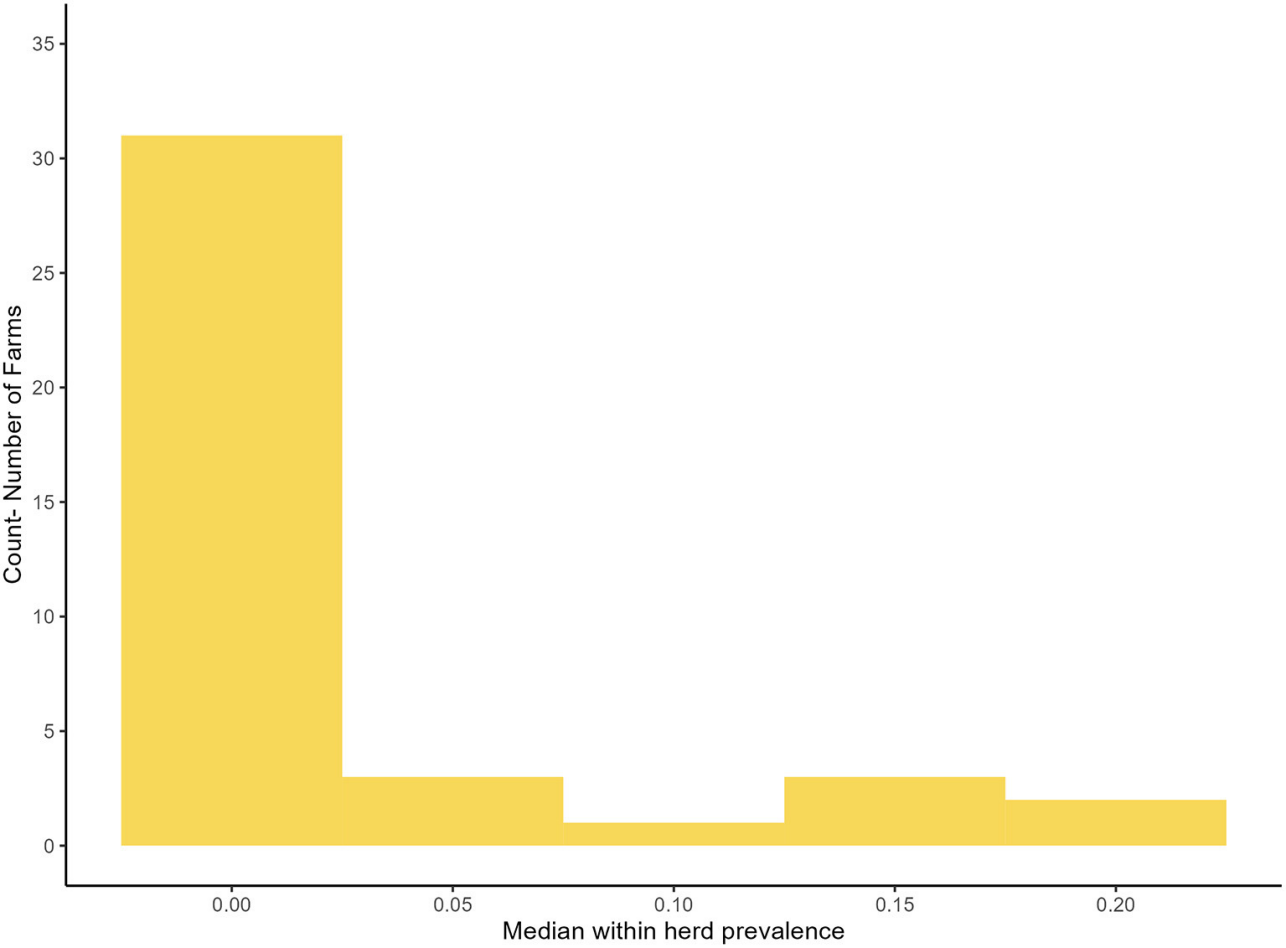
Distribution of the median, true within herd prevalence as produced by the primary model.

In the final model the within-herd prevalence varied from 0% to 22%, the median within herd prevalence was 0% (95% BCI: 0%, 10%). Figure 4 is a histogram of the distribution of within herd prevalence as predicted by the primary model using informative priors, independent tests and a TUS cut off ≥3.

### 3.8. Sensitivity analysis

Table 3 shows the results of each of the alterations made to the models to allow for sensitivity analysis.

#### 3.8.1. Altering priors

The use of uninformed priors for the Se and Sp of each test resulted in an increase in the prevalence in all modeled scenarios, bar one. The change varied from a decrease of 2% prevalence to a maximum increase of 10% of median prevalence. However the 95% BCI in these scenarios remained relatively similar to that of the primary model [95% BCI (1%, 8%)] with the 95% BCI increasing in width the most when uninformative priors were used for TUS [95% BCI (2%, 19%)].

#### 3.8.2. Altering gamma

Altering gamma from (10, 10) to (0.1, 0.1) resulted in an increase in prevalence from 15% [95% BCI (6%, 27%)] to 18% [95% BCI (9%, 27%)] in the model using TUS ≥2 as a cut off. In the primary model there was no change in the median prevalence estimate but a minor increase in the 95% BCI going from (1%, 8%) to (1%, 9%).

#### 3.8.3. Altering TUS cut off

Reducing the TUS score cut off from ≥3 to ≥2 as the case definition resulted in a 11% increase in the median prevalence of BRD in the models using independent tests and informed priors. It also resulted in the 95% BCI becoming wider increasing from (0%, 8%) to (6%, 27%).

#### 3.8.4. Dependency

Test dependency had little effect on either of the primary models with a 1% decrease in conditionally dependent models using TUS ≥2 as the cut off, and a 1% increase in the conditionally dependent model using TUS ≥3 as the cut off. Test dependency had resulted in minor variation in 95% BCI.

## 4. Discussion

To the authors' knowledge, this is the first estimate of calf-level prevalence of BRD in in seasonal calving herds using hierarchical Bayesian latent class techniques. Bayesian latent class techniques have been used previously in the literature to estimate Se and Sp of TUS and CRS (23–25), this method has not been used with the primary focus of estimating the calf-level and within-herd prevalence of BRD. Because of this, comparisons of true BRD prevalence between this work and previously published literature is difficult as the prevalence estimate of 4% accounts for the imperfect Se and Sp of TUS and CRS which is often not the case in other work estimating BRD prevalence.

At the within-herd level the prevalence varied between 0% and 22%. Eleven of the farms visited were negative for BRD on TUS and CRS. This indicates that a low prevalence of BRD is achievable in commercial spring calving herds. Some herds did have a higher prevalence of BRD and a more detailed investigation of the management and environmental factors that influence the prevalence of BRD on those farms will follow on from this work.

The farms used as part of this work were randomly recruited throughout Ireland, which should be considered as a strength of this work. The farms visited were seasonally calving and group-reared calves indoors, reflecting typical calf management in Ireland. The majority of farms were located in Munster, this was a reflection of the demographics of dairy farms in Ireland (48)^2^. The average dairy herd size in Ireland is 92 cows (49), the mean herd size in this work was 145. This is likely a result of the recruitment process as HerdPlus is a paid service, generally used by farmers with larger herds. This may explain why the median group size in this work was larger than that of Barry et al. (36) (11 vs. 18). The relationship between herd size and BRD prevalence is unclear so it is difficult to determine if this had an affect on the prevalence estimate. The sex and breed of sampled calves was biased toward dairy heifers. This was due to the sampling age range. In the majority of farms that were visited, bull calves and beef calves were sold at ~3 weeks of age which would have meant they had left the farm before they were eligible to be sampled; this is typical of most Irish dairy farms (50). The age range of 4–6 weeks was chosen because this has been identified in previous work as the age at which the peak prevalence of BRD is observed (17, 29). This criterion was chosen to decrease the risk of biasing results by sampling of younger populations on given farms. As can be seen from the age range of sampled calves, it was not possible to time all visits so that all 20 calves were between 4 and 6 weeks of age. This was not logistically possible for every farm due to variation in the compactness of farms' calving seasons.

There was a 9% prevalence of CRS cases as defined by a score ≥5 or two scores ≥2 (21). This is lower than the prevalence observed in several studies which used CRS, however direct comparison is difficult due to inconsistency in case definition. Lago et al. (29) reported a prevalence of 14% in Wisconsin, however they used a score ≥6 as a case definition, suggesting if they had used a lower threshold the prevalence observed may have been higher. Medrano-Galarza et al. (28) use a score ≥5 as a case definition and found 17% prevalence of BRD in calves examined. Van Leenen et al. (15) found a prevalence of 20.2% of calves with a score of ≥5. Johnson et al. (3) found an incidence rate of 10.1 cases per 100 calf weeks using a case definition of CRS score ≥5. Calderón-Amor and Gallo (27) found a prevalence of 15.5% of respiratory disease in calves in Chile however the CRS was calculated without inclusion of a rectal temperature score. Initially the authors had hypothesized that there would be a higher prevalence of respiratory disease in the Irish system due to factors such as lack of time and group size. In all cases the reported prevalence of BRD as diagnosed by CRS was higher or similar to prevalence observed in this work. It may be the case that the temperate climate of Ireland is a less conducive environment for respiratory disease compared to work such as Lago et al. (29) and Medrano-Galarza et al. (28) which were undertaken in a microthermal environment. The compact nature of the calving season seen in these herds means that the age range of calves in a given calf house is smaller than an all year round system such as Johnson et al. (3) reducing the potential for older calves to shed pathogens that might cause BRD in younger calves.

In this work 6.5% of calves had a TUS score ≥3 and 17.7% had a TUS ≥2. This level of lung consolidation is similar to other work where Buczinski et al. (18) found a prevalence of 8% in the summer and 15% in the winter of lung consolidation ≥3 cm and Van Leenen et al. (15) found a prevalence of 27.1 % of calves with lung consolidation ≥3 cm. However, direct comparison cannot be made because of the use of a scoring system rather than measuring depth of consolidation.

Consistency in definition of TUS lesions has not been reached within the literature. Some authors, such as Rhodes et al. (17), have used the scoring system as described by Ollivett and Buczinski (14) while others like Cuevas-Gomez et al. (20) and Cramer and Ollivett (51) have made adaptations to this scoring system. Other authors, such as Buczinski et al. (18) and Van Leenen et al. (15), have used various consolidation depths as case definitions for BRD. The lack of consistency makes comparisons difficult. Hence, a consensus needs to be reached as to how TUS results are recorded and interpreted.

Caution must be taken when comparing the prevalence estimates from this work with others, as the method of farm recruitment is not consistent with several studies using convenience samples (3, 15, 27–29). The use of a convenience sample may bias toward farms with higher prevalence or it may bias for farms with lower prevalence depending on the nature of recruitment; if recruitment occurs *via* veterinary practitioners this may bias the estimate higher, if recruitment uses farms with a pre-existing relationship with a university/research organization they may have better management that might bias for a lower BRD prevalence estimate. This makes it difficult to determine whether the prevalence of BRD as determined by CRS/TUS here is lower/similar to other studies with different farming systems. Based on the results from this study it appears that the broad differences in Irish management systems (e.g., seasonal, group-housing vs. all-year round calving, individual housing) does not have a negative impact or may even have a slightly positive impact on BRD prevalence. It is more likely that the individual farm management/facilities within a husbandry system impact BRD prevalence more than the over arching husbandry system itself.

The data gathered here uniquely allowed assignment of BRD cases to one of three different subtypes. Upper respiratory tract disease (URTD) (+ CRS, – TUS), lower respiratory tract disease (LTRD) (– CRS, + TUS) and clinical respiratory disease (CRD) (+ CRS, – TUS). The breakdown of URTD, LRTD, and CRD is likely to be a reflection of the interaction between housing environment, calf immunity and pathogens present on a given farm. When the distribution of the different subtypes from this work was examined two major points emerged. Firstly, a substantial proportion of calves with pulmonary lesions, that may reduce calf performance, are likely to go undiagnosed if only examined for clinical signs using CRS. Of the fifty one calves that were identified with a TUS ≥3, only 14 (19.4%) were identified as cases to be treated using CRS. This means that the majority of calves with pulmonary lesions likely went untreated and so may have had reduced performance. Secondly, when TUS ≥3 was used as the case definition the majority of cases of BRD were classified as URTD. URTD cases were defined as calves that had CRS scores ≥5 or two scores ≥2 but did not have significant lung consolidation. This may have implications for antimicrobial usage as it is not clear currently whether it is of benefit to animals to treat URTD. Future work should investigate response to treatment, prognosis of different BRD subtypes and defining calves with active and non-active BRD.

The epidemiology of the different BRD subtypes has implications for the use of both TUS as a screening method to assess farmer diagnostic Se and clinical scoring as a method by which to choose treatments. However, this was outside of the scope of this work. In the future integration of tools such as activity monitors and automatic feeders may allow for earlier detection of cases and detection of LRTD cases that may have otherwise have gone untreated (52).

In previous publications TUS and CRS have been considered both independent and conditionally dependent (23, 25) when conducting BLCA. However, in recent work cough was the only CRS clinical sign significantly associated with lung consolidation (19). For that reason, we chose a model with independent tests. This was confirmed by the minor variation in prevalence when test dependency was altered in the sensitivity analysis and no change in 95% BCI. Using uninformative priors for both the Se and Sp of CRS and TUS resulted in higher median estimates for prevalence, however the 95% BCI in both cases became wider than the primary model making the model suggesting the model was less identifiable. The methodology by which the priors were chosen for this work is robust and should be considered more reliable than the prevalence estimated using uninformative priors. However, one must conclude that in this case the test priors did affect the prevalence estimates, this is likely due to the number of calves enrolled and the hierarchical structure of the model. The number of calves and model structure is also likely to have contributed to the broad confidence intervals seen around the prevalence estimate in some of the models this is a weakness of this work. However, even with wide 95% BCI, the majority of the models still produced estimates and confidence intervals that are below or similar to estimates for other production systems.

As expected, the choice of TUS case threshold affected the true prevalence estimate. BRD occurs on a continuous spectrum, and so choosing a clinically relevant cut off is difficult. In the case of a fixed target condition, the effect of changing the case definition threshold is to increase or decrease the Se and Sp of a test. In latent class analysis the latent state is to some extent dependent on the tests, for example Nielsen et al. (53) showed true prevalence estimates in one cluster reduced form 56% to 2.3% when the ELISA cut-off was changed when testing bulk milk samples for *Mycoplasma bovis*. In this case we chose to vary the TUS positive definition because there is still some uncertainty in the literature around the positivity threshold. Cramer and Ollivett (51) showed that a TUS ≥2 and Rhodes et al. (17) found that a TUS ≥3 was associated with a decreased growth rate. The work conducted by Rhodes et al. (17) was conducted in an Irish calf population and so is more applicable to this model hence a TUS ≥3 was used. However, a score 2 still indicates a disease process is occurring and so the authors felt varying the threshold would be a useful exercise and provide greater context for readers. When TUS ≥2 was considered the case definition, the median estimates of BRD prevalence from the model increased while the 95% BCI also became wider, this should be considered a worst case scenario BRD prevalence estimate and due to the wide BCI the authors would have less confidence in this estimate. In the work sighted case definitions were based on changes in growth rate. Because growth rates changes are an indirect effect of BRD using them do justify a particular cut off may have effect of biasing treatment recommendations.

There were several limitations in this work: Firstly, we modeled within herd prevalence across both years with a single distribution. However, since the incidence (and therefore the prevalence) of BRD is impacted by environmental factors, the prevalence distributions might have been different across both years. To investigate, we repeated the analysis by modeling the prevalence distribution in each year separately. However, we found no difference in the median estimates across years and therefore decided to maintain the simpler model (see Supplementary material). Secondly, it was not possible to for the TUS assessor to be blinded to the results of the CRS scoring process since both occurred at the same time. However, the lack of agreement between the results of both tests suggests that this is unlikely to have been an issue in our study, TUS is the more objective of the two scoring systems and so is less likely to be influenced by results as compared to CRS. Thirdly, due to the cross-sectional nature of this work it was not possible to determine if the cases were active in nature, this would be useful as a distinction between active and non-active infections informs treatment recommendations and management practices. Instead, we focused on the BRD subtypes which is still useful in that it gives us a more detailed understanding of the epidemiology of BRD in Ireland. Finally, we did not factor in treatment records or vaccination programs implemented on thee farms. The use of vaccination, as well as the ability of the farmer to diagnose cases and implement effective therapy may account for some of the variation in farm-level prevalence of BRD. However, the goal of this research was to estimate the true prevalence of BRD. Our sample is expected to consist of a range of vaccination and detection methods which we assume are reflective of dairy herds nationally.

## 5. Conclusion

We estimated the BRD prevalence to be 4% using a Bayesian approach to account for the lack of a gold standard antemortem test. The prevalence of BRD was lower or similar to that seen in other systems and regions. In future work investigating BRD prevalence, a Bayesian approach using two imperfect tests should be taken to allow for comparison of prevalence between different calf rearing systems. In addition, we found variation in the prevalence of different BRD subtypes with upper respiratory tract disease appearing to be more common. Future work should investigate the epidemiology of these different disease subtypes and what farm and pathogen-level risk factors influence them as well as treatment protocols.

## Data availability statement

The raw data supporting the conclusions of this article will be made available by the authors, without undue reservation.

## Ethics statement

The animal study was reviewed and approved by Animal Research Ethics Committee University College Dublin. Written informed consent was obtained from the owners for the participation of their animals in this study.

## Author contributions

CM and JM contributed to conception and design of the study. JD collected and managed the data and wrote the first draft of the manuscript. JD and CM performed the statistical analysis. JM, CM, and JD wrote sections of the manuscript. All authors contributed to manuscript revision, read, and approved the submitted version.

## Footnotes

^1^ https://www.met.ie/climate/climate-of-ireland

^2^ https://www.cso.ie/en/releasesandpublications/ep/p-coa/censusofagriculture2020-preliminaryresults/farmstructure/
