# Supplementary material for: Prevalence of respiratory disease in Irish preweaned dairy calves using hierarchical Bayesian latent class analysis
Source: Front Vet Sci. 2023 Apr 13;10:1149929. doi: 10.3389/fvets.2023.1149929 (PMC10133517; doi:10.3389/fvets.2023.1149929)
Supplement: Supplementary file 1 [file Data_Sheet_1.docx]

**Supplemental materials:**

Inclusion criteria:

The information required for inclusion were; 1.) Test must be conducted in calves on farm; hospitalized calves were considered a biased population, 2.) A statistical framework must be used that accounted for the lack of a gold standard test (i.e. using thoracic ultrasound results as a reference was not considered adequate), 3.) BRD case definition must be clearly stated, 4.) The sensitivity and specificity must be calculated for the Wisconsin respiratory scoring system as described earlier in materials and methods of this paper.

**Table 1.** Search terms, dates and number of hits for each of the search conducted to estimate Se and Sp of thoracic ultrasound and Wisconsin respiratory score

| **Search String** | **Database** | **Date Searched** | **No. Hits** |
| --- | --- | --- | --- |
| (("BRD" OR "bovine respiratory disease" ) AND ("clinical scoring system" OR "Wisconsin clinical score" OR "clinical score")) AND (sensitivity OR specificity OR accuracy) | PubMed | 30/11/2022 | 21 |
| (("BRD" OR "bovine respiratory disease") AND ("clinical scoring system" OR "Wisconsin clinical score" OR "clinical score") AND (sensitivity OR specificity OR accuracy)) | CABI Direct | 30/11/2022 | 16 |
| (("BRD" OR "bovine respiratory disease") AND ("thoracic ultrasound" OR "TUS")) AND (sensitivity OR specificity OR accuracy) | PubMed | 30/11/2022 | 16 |
| (("BRD" OR "bovine respiratory disease") AND ("thoracic ultrasound" OR "TUS") AND (sensitivity OR specificity OR accuracy)) | CABI Direct | 30/11/2022 | 7 |

**Table 2.** Thoracic ultrasound scoring sensitivity and specificity results from a systematic literature review

| **Authors** | **Year** | **Se** | | | **Sp** | | |
| --- | --- | --- | --- | --- | --- | --- | --- |
|  |  | **Median** | **Lower** | **Upper** | **Median** | **Lower** | **Upper** |
| Decaris *et al.*(1) | 2022 | 0.67 | 0.57 | 0.78 | 0.87 | 0.82 | 0.92 |
|  |  | 0.60 | 0.47 | 0.73 | 0.85 | 0.80 | 0.90 |
| Berman et al.(2) | 2019 | 0.90 | 0.61 | 1.00 | 0.77 | 0.73 | 0.81 |
| Buczinski *et al.* (3) | 2015 | 0.79 | 0.66 | 0.91 | 0.94 | 0.88 | 0.98 |
|  |  | 0.78 | 0.65 | 0.90 | 0.94 | 0.89 | 0.98 |
|  |  | 0.7 | 0.55 | 0.87 | 0.94 | 0.89 | 0.98 |
|  |  | 0.71 | 0.58 | 0.84 | 0.95 | 0.86 | 0.99 |
| Buczinski *et al.*(4) | 2016 | 0.77 | 0.6 | 0.89 | 0.93 | 0.87 | 0.97 |
|  |  | 0.71 | 0.53 | 0.85 | 0.83 | 0.75 | 0.9 |
|  | Mean | 0.732 | 0.576 | 0.86 | 0.888 | 0.83 | 0.932 |

**Table 3.** Clinical scoring sensitivity and specificity results from a systematic literature review

| **Authors** | **Year** | **Se** | | | **Sp** | | |
| --- | --- | --- | --- | --- | --- | --- | --- |
|  |  | **Median** | **Lower** | **Upper** | **Median** | **Lower** | **Upper** |
| Decaris *et al.*(1) | 2022 | 0.75 | 0.61 | 0.88 | 0.82 | 0.77 | 0.87 |
|  |  | 0.78 | 0.65 | 0.9 | 0.82 | 0.76 | 0.88 |
| Berman *et al.* (2) | 2019 | 0.69 | 0.4 | 0.97 | 0.95 | 0.92 | 0.97 |
|  |  | 0.48 | 0.12 | 0.91 | 0.93 | 0.9 | 0.95 |
|  |  | 0.7 | 0.4 | 0.97 | 0.95 | 0.92 | 1 |
|  |  | 0.7 | 0.3 | 0.98 | 0.91 | 0.87 | 0.94 |
|  |  | 0.66 | 0.31 | 0.97 | 0.99 | 0.98 | 1 |
| Buczinski *et al.* (3) | 2015 | 0.62 | 0.48 | 0.76 | 0.74 | 0.65 | 0.83 |
|  |  | 0.66 | 0.55 | 0.76 | 0.77 | 0.7 | 0.84 |
|  |  | 0.61 | 0.48 | 0.74 | 0.78 | 0.68 | 0.87 |
|  |  | 0.63 | 0.48 | 0.76 | 0.76 | 0.66 | 0.85 |
|  | Mean | 0.657 | 0.401 | 0.867 | 0.856 | 0.792 | 0.907 |

| **Case Definition** | **Gamma Prior** | **TUS- Prior Beta** | | **CRS- Prior Beta** | | **Independent Models (Median (95% BCI))** | | | | **Co-dependent Models (Median (95%BCI))** | | | |
| --- | --- | --- | --- | --- | --- | --- | --- | --- | --- | --- | --- | --- | --- |
|  |  |  |  |  |  | **TUS** | | **CRS** | | **TUS** | | **CRS** | |
|  |  | **Se** | **Sp** | **Se** | **Sp** | **Se** | **Sp** | **Se** | **Sp** | **Se** | **Sp** | **Se** | **Sp** |
| **TUS ≥ 2** | (10, 10) | (27.6, 10.7) | (89.3, 8.4) | (9.8, 5.6) | (138.8, 18.5) | 0.62 (0.49, 0.78) | 0.91 (0.87, 0.95) | 0.38 (0.25, 0.55) | 0.95 (0.93, 0.97) | 0.66 (0.55, 0.77) | 0.90 (0.87, 0.94) | 0.44 (0.31, 0.58) | 0.96 (0.94, 0.97) |
|  | (10, 10) | (1, 1) | (1, 1) | (9.8, 5.6) | (138.8, 18.5) | 0.47 (0.32, 0.64) | 0.91 (0.85, 0.96) | 0.35 (0.21, 0.55) | 0.96 (0.94, 0.98) | 0.52 (0.29, 0.72) | 0.87 (0.82, 0.93) | 0.47 (0.27, 0.69) | 0.95 (0.93, 0.97) |
|  | (10, 10) | (27.6, 10.7) | (89.3, 8.4) | (1, 1) | (1, 1) | 0.51 (0.41, 0.61) | 0.93 (0.89, 0.96) | 0.32 (0.22, 0.44) | 0.99 (0.98, 1.00) | 0.65 (0.53, 0.75) | 0.93 (0.89, 0.96) | 0.44 (0.31, 0.58) | 0.99 (0.98, 1.00) |
|  | (1,1) | (27.6, 10.7) | (89.3, 8.4) | (9.8, 5.6) | (138.8, 18.5) | 0.62 (0.49, 0.76) | 0.91 (0.88, 0.95) | 0.38 (0.26, 0.53) | 0.95 (0.93, 0.97) | 0.67 (0.56, 0.77) | 0.90 (0.87, 0.94) | 0.45 (0.33, 0.57) | 0.96 (0.94, 0.97) |
|  | (0.1, 0.1) | (27.6, 10.7) | (89.3, 8.4) | (9.8, 5.6) | (138.8, 18.5) | 0.62 (0.50, 0.76) | 0.92 (0.88, 0.95) | 0.38 (0.26, 0.52) | 0.95 (0.93, 0.97) | 0.67 (0.57, 0.77) | 0.90 (0.87, 0.94) | 0.46 (0.34, 0.58) | 0.96 (0.94, 0.97) |
| **TUS ≥ 3** | (10, 10) | (27.6, 10.7) | (89.3, 8.4) | (9.8, 5.6) | 0.64 (0.47, 0.8) | 0.66 (0.49, 0.82) | 0.96 (0.94, 0.97) | 0.61 (0.42, 0.80) | 0.93 (0.90, 0.95) | 0.61 (0.45, 0.78) | 0.96 (0.94, 0.98) | 0.60 (0.42, 0.77) | 0.93 (0.91, 0.96) |
|  | (10, 10) | (1, 1) | (1, 1) | (9.8, 5.6) | (138.8, 18.5) | 0.30 (0.16, 0.5) | 0.96 (0.94, 0.98) | 0.53 (0.34, 0.73) | 0.95 (0.93, 0.97) | 0.31 (0.11, 0.52) | 0.96 (0.93, 0.98) | 0.60 (0.39, 0.80) | 0.95 (0.93, 0.97) |
|  | (10, 10) | (27.6, 10.7) | (89.3, 8.4) | (1, 1) | (1, 1) | 0.49 (0.33, 0.74) | 0.96 (0.94, 0.98) | 0.62 (0.39, 0.88) | 0.97 (0.93, 1.00) | 0.48 (0.37, 0.61) | 0.96 (0.95, 0.98) | 0.67 (0.51, 0.82) | 0.98 (0.95, 1.00) |
|  | (1,1) | (27.6, 10.7) | (89.3, 8.4) | (9.8, 5.6) | (138.8, 18.5) | 0.66 (0.49, 0.82) | 0.96 (0.94, 0.97) | 0.61 (0.43, 0.80) | 0.93 (0.90, 0.95) | 0.59 (0.44, 0.76) | 0.96 (0.94, 0.98) | 0.61 (0.44, 0.77) | 0.93 (0.91, 0.96) |
|  | (0.1, 0.1) | (27.6, 10.7) | (89.3, 8.4) | (9.8, 5.6) | (138.8, 18.5) | 0.65 (0.47, 0.81) | 0.96 (0.94, 0.97) | 0.61 (0.43, 0.79) | 0.93 (0.91, 0.95) | 0.57 (0.42, 0.73) | 0.96 (0.94, 0.97) | 0.61 (0.45, 0.77) | 0.94 (0.91, 0.96) |

**Table 4.** Sensitivity and specificity estimates for each model:

**Table 5.** Prevalence of BRD when each year was modelled separately

| **Year** | **Prevalence (Median (95% BCI))** | **Alpha (Median (95% BCI))** | **Mu (Median (95% BCI))** |
| --- | --- | --- | --- |
| **1** | 0.03 (0.00, 0.08) | -2.65 (-4.07, -1.24) | 0.52 (0.18, 0.97) |
| **2** | 0.04 (0.00, 0.13) | -1.81 (-3.74, 0.01) | 0.30 (0.03, 0.73) |

**Reference**

1. Decaris N, Buczinski S, Tárdon DIC, Camargo L, Schllemer NR, Hagen SCF, et al. Diagnostic accuracy of Wisconsin and California scoring systems to detect bovine respiratory disease in preweaning dairy calves under subtropical environmental conditions. J Dairy Sci. 2022 Sep 1;105(9):7750–63.

2. Berman J, Francoz D, Dufour S, Buczinski S. Bayesian estimation of sensitivity and specificity of systematic thoracic ultrasound exam for diagnosis of bovine respiratory disease in pre-weaned calves. Prev Vet Med. 2019 Jan 1;162:38–45.

3. Buczinski S, L Ollivett T, Dendukuri N. Bayesian estimation of the accuracy of the calf respiratory scoring chart and ultrasonography for the diagnosis of bovine respiratory disease in pre-weaned dairy calves. Prev Vet Med. 2015 May 1;119(3):227–31.

4. Buczinski S, Ménard J, Timsit E. Incremental Value (Bayesian Framework) of Thoracic Ultrasonography over Thoracic Auscultation for Diagnosis of Bronchopneumonia in Preweaned Dairy Calves. J Vet Intern Med. 2016;30(4):1396–401.
